# Interleukin-1 Receptor-Associated Kinase 4 Deficiency in a Greek Teenager

**DOI:** 10.1155/2020/8846827

**Published:** 2020-10-08

**Authors:** Panagiota Karananou, Anastasia Alataki, Efimia Papadopoulou-Alataki

**Affiliations:** 4th Department of Paediatrics, School of Medicine, Faculty of Health Sciences, Aristotle University of Thessaloniki, Papageorgiou General Hospital, Thessaloniki, Greece

## Abstract

**Background:**

Human interleukin- (IL-) 1 receptor-associated kinase 4 (IRAK-4) deficiency is a recently described primary immunodeficiency. It is a rare, autosomal recessive immunodeficiency that impairs toll/IL-1R immunity, except for the toll-like receptor (TLR) 3- and TLR4-interferon alpha (IFNA)/beta (IFNB) pathways. *Case Report*. We report the first patient in Greece with IRAK-4 deficiency. From the age of 8 months, she presented with recurrent infections of the upper and lower respiratory tract and skin abscesses. For this, she had been repeatedly hospitalized and treated empirically with intravenous antibiotics. No severe viral, mycobacterial, or fungal infections were noted. Her immunological laboratory evaluation revealed low serum IgA and restored in subsequent measurements; normal IgG, IgM, and IgE; and normal serum IgG subclasses. Peripheral blood immunophenotyping by flow cytometry and dihydrorhodamine (DHR) test revealed normal counts. She was able to make functional antibodies against vaccine antigens, including tetanus and diphtheria. She was administered with empirical IgG substitution for 5 years until the age of 12 years, and she has never experienced invasive bacterial infections so far. DNA analysis revealed a heterozygous variant in the patient: c.823delT (p.S275fs^*∗*^13 at protein level) in the IRAK4 gene.

**Conclusions:**

The importance of clinical suspicion is emphasized in order to confirm the diagnosis by IRAK4 gene sequencing and provide the appropriate treatment for this rare primary immunodeficiency, as soon as possible.

## 1. Introduction

Human interleukin- (IL-) 1 receptor-associated kinase 4 (IRAK-4) deficiency is a recently described primary immunodeficiency (PID) [1]. It is a rare, autosomal recessive immune deficiency that impairs toll/IL-1R immunity, except for the toll-like receptor (TLR) 3- and TLR4-interferon alpha (IFNA)/beta (IFNB) pathways [2]. Toll-like receptors and interleukin-1 receptors are essential for the recognition of microbes and signaling pathway through MyD88 (myeloid differentiation molecule) that recruits IRAK-4. IRAK-4 deficiency highly predisposes to bacterial infections [3].

Picard et al., in 2003, was the first to describe a case series of 3 unrelated children with recurrent infections caused only by extracellular pyogenic bacteria and poor inflammatory response [4]. IRAK-4 deficiency (OMIM gene606883 and OMIM phenotype 607676) is described by the International Union of Immunological Societies Committee in the major PID group: defects in intrinsic and innate immunity as a TIR signaling pathway deficiency with bacterial infections [5]. Until now, more than 50 patients were studied and reported to suffer from IRAK-4 deficiency worldwide [6].

## 2. Clinical Case

We report the case of a 17-year-old female patient, the only child of healthy unrelated parents with no family history of recurrent or severe infections, autoimmune disease, or lymphoma. She had an uncomplicated perinatal history, with a normal interval for umbilical cord separation (7 days). The girl was fully immunized, including the conjugated *pneumococcal* vaccine, as well as *measles*, *mumps*, *rubella*, and *varicella*, and BCG live vaccinations with no adverse effects.

At the age of 8 months, she presented to the hospital with bronchiolitis. Since then, she suffered from recurrent respiratory tract infections (sinusitis, mastoiditis, otitis, and pharyngitis) every 1–6 months until the age of 3 years. Thereafter, she also developed skin abscesses (*Staphylococcus aureus* was detected) treated by appropriate antibiotic therapy within the hospital. From 3 years until 5 years, she had been hospitalized twice per year and treated empirically with intravenous antibiotics (ampicillin and/or cephalosporins). At the age of 5 years, she underwent adenoidectomy and tonsillectomy and received antibiotic prophylaxis for 6 months without recession of the infections (Table 1). No severe viral, mycobacterial, or fungal infections were noted. She was also diagnosed with intellectual disability within autism spectrum.

Her immunological laboratory evaluation performed at 6 years of age revealed low serum IgA, that has been restored in subsequent measurements; normal IgG, IgM, and IgE; and normal serum IgG subclasses (except from IgG4, also restored in subsequent measurements). Peripheral blood immunophenotyping by flow cytometry revealed normal counts. The circulating monocyte counts and neutrophil counts are normal. The dihydrorhodamine (DHR) test for the estimation of the phagocytic function was normal for her age (Table 2). She was able to make functional antibodies against vaccine antigens, including tetanus and diphtheria.

At the age of 7 years, she started being treated with prophylactic subcutaneous immunoglobulin therapy. The treatment lasted 5 years resulting in significant reduction of infections (Table 1). The patient was studied by genetic analysis (sequencing of genomic DNA). The parents could not be investigated. Since the age of 12 years, she is no longer suffering from infections, she is free of treatment, but she still presents with learning difficulties and behavioral problems.

## 3. Genetic Analyses

Whole exome sequencing (WES) was performed. DNA-analysis was done using Otogenetics Corporation (USA), and identification of pathogenic and disease-causing variants was conducted using the Ingenuity Variant Analysis software (Qiagen). Sequencing of IRAK4 showed that a change in a single nucleotide base occurred in five transcript variants of the gene. In particular, two of the mRNA variants had a heterozygous deletion of thymidine 823 of IRAK4 (c.823delT), and three variants had a heterozygous deletion of thymidine 451 of IRAK4 (c.451delT) (Table 3). The first two transcript variants encode isoform A of the IRAK-4 protein, whilst the other three encode isoform B (based on NCBI).

**Figure 1:**
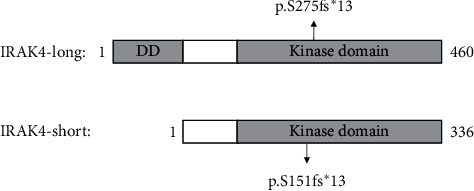
Schematic representation of two protein isoforms of IRAK-4 with the reported protein changes. The IRAK-4 protein contains an *N*-terminal death domain (DD), a hinge domain (labelled in white), and the kinase domain. The full-length DNA of the IRAK4 gene encodes the IRAK4-long protein consisting of 460 amino acids, while the alternative spliced gene encodes the IRAK4-short protein of 336 amino acids.

Isoform A (IRAK4-long) induces the activation of NF-*κ*B through MyD88 binding, and it is associated with diseases, such as primary immunodeficiency diseases and cancer [7, 8]. In contrast, isoform B (IRAK4-short) is less efficient at activating NF-*κ*B and the innate immune pathway and is preferentially expressed in normal tissues [8] (Figure 1). As such, c.823delT (p.S275fs^*∗*^13 at protein level) is considered a heterozygous mutated disease-causing variant of the IRAK4 gene in this patient and has been previously reported [7].

## 4. Discussion

IRAK-4 protein is the fourth member of the IRAK family (IRAK-1, IRAK-2, and IRAK-3/M) that, together with MyD88 (a key cytosolic adapter molecule), is found to play a pivotal role in the signaling pathway that is involved in the early recognition of pathogens and the initiation of the cascade of the inflammatory response [3].

**Figure 2:**
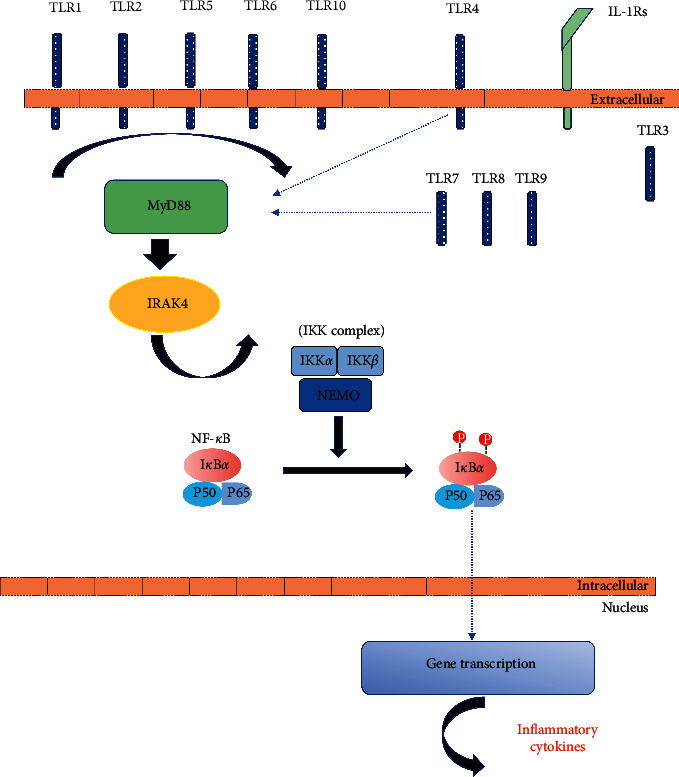
MyD88 and IRAK-4-mediated signaling pathway.

The TIR superfamily (TLRs/IL-1Rs) depends on MyD88 and IRAK-4 signaling for its regulation of gene transcription. After MyD88 is activated, it provides a bridge using its TIR domain from TLRs and interleukin-1 receptors (IL-1Rs) to the IRAK complex. MyD88 forms an oligomer and then recruits IRAK-4 to the receptor, triggering the activation of the I*κ*B kinase (IKK) complex. The activation of the IKK complex promotes the phosphorylation and degradation of I*κ*B*α* and the translocation of NF-*κ*B to the nucleus leading to the production of inflammatory cytokines [9, 10] (Figure 2).

Human IRAK4 gene maps to chromosome 12q12, contains 13 exons, and provides instructions for making a protein that plays an important role in stimulating the innate immune response against infections. IRAK-4 deficiency is caused by mutations in the IRAK4 gene that lead to the production of a nonfunctional protein [9].

Clinically, affected IRAK-4-deficient patients suffer from recurrent infections caused by pyogenic bacteria, mainly Gram-positive, and tend to develop weak or delayed systemic signs of inflammation (e.g., fever) and minimal change in inflammatory markers (e.g., C-reactive protein) [3]. Most of these patients develop their first invasive infection before the age of 2 years and present with peripheral (e.g., skin infection, cellulitis, furuncles, and otitis media) and/or invasive bacterial diseases (e.g., meningitis, arthritis, septicemia, and visceral abscess) [10]. Τhe most common Gram-positive bacteria associated with IRAK-4 deficiency are *Streptococcus pneumoniae* and *Staphylococcus aureus* followed by Gram-negative bacteria, such as *Pseudomonas aeruginosa, Neisseria,* and *Shigella* in a less frequent rate [11]. None of the patients reported had severe viral, parasitic, or fungal disease. It is remarkable that all life-threatening infections occur during early infancy and that there is an overall trend towards improvement of their severity and frequency after the teenage years. Prophylactic antibiotic treatment and vaccinations against pyogenic bacteria, as well as immunoglobulin replacement starting early in life until teenage, are suggested as supportive treatment [3].

Our patient is the first patient in Greece who has been identified with IRAK-4 deficiency, and we report this case to point out the diagnosis challenge that can raise. The onset of her infections was before the age of 2 years as previously reported [1, 10, 12]. She presented with recurrent noninvasive pyogenic bacterial infections of the respiratory tract and skin which were successfully and promptly treated with intravenous or oral antibiotics, that comes in accordance with the existing literature [1]. Her immunological investigations were normal, and she produced adequate antibodies against specific vaccines antigens. As recommended by Picard et al., our patient was administered with empirical IgG substitution until the age of 12 years, a prophylaxis that seems to have been beneficial, since she has never experienced bacterial infections so far [1].

The patient's DNA analysis revealed the change of c.823delT and p.S275fs at the protein level in heterozygosity. This change affecting isoform A of IRAK-4 could alter the kinase activity since it is located in the kinase domain of the protein resulting in crucial impairment of IRAK-4 signaling pathway, leading to disease [12]. Therefore, the heterozygous mutation could result in the phenotype of our patient.

Although the prognosis of IRAK-4 deficiency is poor in early childhood, with a mortality rate of 30%–43% before the age of 8 years, it improves substantially with age [1, 2, 12, 13]. This finding is remarkable in the field of primary immunodeficiencies, that always deteriorate with time, and may be due to the acquisition of humoral immunity and immunologic memory with age (adaptive antigen specific T- and B- lymphocyte responses) [5, 9, 10].

In conclusion, IRAK-4 deficiency should be considered in differential diagnosis of children presenting with severe infections whose immunological evaluations, including those for innate immunity, are unremarkable. The importance of clinical suspicion is emphasized in order to confirm the diagnosis by the IRAK4 gene sequencing and provide the appropriate treatment for this rare primary immunodeficiency, as soon as possible.

## Figures and Tables

**Table 1:** Summary of the patient's infections and therapy.

Age	Infection	Treatment
8 months old	Bronchiolitis	Intravenous ampicillin-nebulized salbutamol-O_2_
9 months old	Laryngitis	Nebulised adrenaline
12 months old	Bronchiolitis	Nebulised salbutamol-ipratropium bromide
14 months old	Acute otitis media	Intravenous ampicillin-sulbactam
17 months old	Bronchiolitis	Nebulised salbutamol-fluticasone
19 months old	Pharyngitis	Oral amoxycillin
21 months old	Acute otitis media	Intravenous cephalosporin
2 years old	Pharyngitis	Oral cephalosporin
2.5 years old	Sinusitis-mastoiditis	Intravenous cephalosporin
3 years old	Skin abscesses-MSSA	Intravenous oxacillin
3.5 years old	Sinusitis	Intravenous cephalosporin
4 years old	Tonsilitis	Intravenous cephalosporin
4.5 years old	Skin abscess-MRSA	Intravenous vancomycin
5 years old		Adenoidectomy-tonsillectomy
6 years old	Acute otitis media	Intravenous ampicillin-sulbactam
7 years old		Subcutaneous immunoglobulin treatment

**Table 2:** Summary of the patient's immunological profile.

Parameter	Normal range	Baseline^*∗*^	17 years old
Blood hemoglobin (g/dL)	12–15.4	13	13.5
Blood platelets (G/L)	150–400	219	357
Blood leukocytes (mm^3^)	3900–11.000	10.400	10.200
Blood neutrophils (%)	40–75	68.6	59.8
Blood lymphocytes (%)	19–48	23.7	30.2
Monocytes (%)	3.4–9	5.3	6.97
Eosinophils (%)	0–7	1.9	2.12
Basophils (%)	0–1.5	0	0.8
IgA (mg/dl)		**26.50** (NR: 60–220)	72.40 (NR: 70–400)
IgG (mg/dl)		717 (NR: 600–1300)	784 (NR: 700–1600)
IgM, (mg/dl)		50.80 (NR: 40–160)	61.60 (NR: 40–230)
IgE (IU/ml)		61.30 (NR: <52)	54.20 (NR: <378)
IgG1 (mg/dl)		633 (NR: 561–1100)	556 (NR: 405–1011)
IgG2 (mg/dl)		89.80 (NR: 86–355)	164 (NR: 169–426)
IgG3 (mg/dl)		53 (NR: 31–100)	27 (NR: 11–85)
IgG4 (mg/dl)		**15.60** (NR: 20–117)	42.30 (NR: 3–201)
CD2 T cells (%)	75.9–84.9	75.3	75.9
CD3^+^ T cells (%)	55–78	69	71.1
CD3^+^/CD4^+^ (%)	27–53	52	49.1
CD3^+^/CD8^+^ (%)	19–34	12.5	15.9
CD3^−^/CD16^+^CD56^+^ NK (%)	4–26	18.9	4.6
CD19^+^ B cells (%)	10–31	10.2	20.1
CD23 (%)		N/D	13.4
CD4^+^/CD8^+^ ratio	0.9–2.6	4.2	3.08
CD3^+^/*γδ*		18.5	N/D
CD3^+^/*αβ*		76.1	N/D
DHR_1,2,3_ (%)		97.3	97.5

^*∗*^Baseline, first investigation of the patient. NR, normal ranges, DHR_1,2,3_, Dihydrorhodamine test. The IgA and IgG4 values are in bold to suggest that they are below the normal range.

**Table 3:** Whole exome sequencing analysis.

Gene	c-DNA nucleotide change	Genomic location	Protein change	Variant classification	RS number	Disease	Inheritance	Status
IRAK4 (OMIM 606883)	NM_001114182.2: c.823delT, NM_016123.3: c.823delT, NM_001145256.1: c451delT, NM_001145257.1: c451delT, NM_001145258.1: c.451delT (ingenuity)	chr12: g 44171539	p.S275fs^*∗*^13, p.S151fs^*∗*^13 (ingenuity)	Pathogenic (ingenuity)	—	IRAK4 deficiency (OMIM 607676)	Autosomal recessive (AR)	Heterozygous

## Data Availability

The data used to support the findings of this study are available from the corresponding author upon request.
